# Identifying microRNA Expression Alterations in Erythrocytes, Lymphocytes, and Monocytes During Severe COVID-19

**DOI:** 10.32607/actanaturae.27610

**Published:** 2026

**Authors:** A. A. Artamonov, Yu. V. Nikitin, A. A. Velmiskina, S. V. Mosenko, V. S. Shimansky, A. Yu. Asinovskaya, S. V. Apalko, N. N. Sushentseva, A. M. Ivanov, S. G. Scherbak, K. A. Kondratov

**Affiliations:** S. M. Kirov Military Medical Academy, St. Petersburg, 194044 Russia; City Hospital No. 40, St. Petersburg, 197706 Russia; St. Petersburg State University, St. Petersburg, 199034 Russia

**Keywords:** miRNA, severe COVID-19, erythrocytes, monocytes, lymphocytes, sRNA, NGS

## Abstract

A significant portion of the fatal outcomes during COVID-19 have been traced mainly to cytokine storm, the uncontrolled hyperactivation of the immune system. During a SARS-CoV-2 infection, the blood plasma levels of microRNAs (miRNAs), a class of short regulatory RNAs, get significantly changed. However, it still remains unknown how the levels and characteristics of these molecules are altered in various blood cells during severe COVID-19. The aim of this research was to compare the microRNA levels in erythro cytes, monocytes, and lymphocytes in normal blood cells and those in patients with severe COVID-19-induced by cytokine storm. Erythrocytes and monocytes (five healthy donors and five patients with severe COVID-19) and lymphocytes (four healthy donors and four patients with severe COVID-19) were obtained by fluores cence-activated cell sorting. RNA was isolated from the obtained cells, and next-generation short RNA se quencing was performed. Both the known miRNAs and the novel miRNAs whose expression had changed in severe COVID-19 were analyzed and identified. In the erythrocytes, seven miRNAs had changed expres sions (five downregulated; two upregulated); all 13 miRNAs were upregulated in lymphocytes; in monocytes, 11 miRNAs were downregulated and three miRNAs were upregulated. An analysis of the novel miRNAs showed that three, previously unknown miRNAs, were downregulated in lymphocytes and one was upregu lated. In monocytes and erythrocytes, no novel, differentially expressed miRNAs were detected. Additionally, we analyzed the signaling pathways altered by miRNAs by performing a miRNA enrichment analysis (MIEAA) using the Gene Ontology miRNA target database (miRTarBase). We observed that in lymphocytes, four pathways were significantly (Q-value < 0.05) enriched and 339 were depleted; in monocytes, 118 path ways were enriched and six were depleted. No significantly altered signaling pathways were detected in erythrocytes.

## INTRODUCTION

COVID-19 is a complex disease that alters the expres sion profiles of many molecules in blood cells [1]. The vast majority of studies in this field compare the ex pression profiles of protein-synthesizing genes. Non coding RNAs – long non-coding RNAs, piRNAs [2], and especially miRNAs – have previously been shown to play a key regulatory role. In particular, miRNAs are key immune response regulators, influencing the maturation, proliferation, differentiation, and activa tion of immune cells, antibody production, as well as the release of inflammatory mediators [3]. The defin ing observations about changes in miRNA levels are typically made in blood plasma and serum. A whole spectrum of these molecules has been acknowledged as markers of severe COVID-19 [4]. However, it re mains unclear from which cells the miRNAs are re leased into plasma. Of particular importance is also the changes in the intracellular signaling processes that occur under the influence of noncoding RNAs. Changes in long non-coding RNAs in monocytes and lymphocytes during COVID-19 have been demonstrated earlier [5, 6]. Nevertheless, the influence of actively synthesizing cells is not the only factor that impacts the plasma miRNA levels. The miRNA pro f iles in erythrocytes were shown to undergo chang es during sickle cell anemia [7], paroxysmal noctur nal hemoglobinuria [8], as well as Parkinson’s disease [9]. Additionally, it has been established that these cells secrete extracellular vesicles that carry numer ous miRNAs modulating the immune response [10]. The aim of this research was to compare the miRNA expression profiles in erythrocytes, lymphocytes, and monocytes from control donors and patients with se vere COVID-19, as well as evaluate how the signaling pathways in these cells are altered.

## EXPERIMENTAL

**Patients, blood collection and f luorescence cell sorting**

Blood from five control donors (four males and one female aged 29–73 years) and five patients with se vere COVID-19 (four males and one female aged 53 76 years) was collected from the median cubital vein via venipuncture. The clinical data of the patients with severe COVID-19 are presented in Table 1 and the Supplementary Materials. Informed consent was secured from all involved in the study. This study was conducted in compliance with the guidelines of the Declaration of Helsinki and approved by the Ethics Committee of City Hospital No. 40 (Sestroretsk, St. Petersburg, Russia) on May 18, 2020 (protocol No. 171).

**Table 1:** Clinical data of severe COVID-19 patients

Patient	Sex	Age, years	Interleukin-6, pg/mL	CRP (C-reactive protein), mg/L	The patient’s condition at the time of blood sampling
1	male	53	34	106.7	Severe
2	male	57	204	45.1	Severe
3	male	73	62	105.7	Severe
4	female	75	24	58.9	Moderate
5	male	76	2398	10.2	Moderate

All the cell types were sorted on a MoFlo Astrios EQ cell sorter (Beckman Coulter, USA). For eryth rocyte sorting, 2 µL of whole blood was dissolved in 100 µL of PBS and stained with antibodies specific to CD235, CD45, and CD41. After staining, 5 × 10^6^ CD235+ events were sorted. The precipitate was fro zen in dry ice. To sort lymphocytes and monocytes, 100 µL of whole blood was treated with 1 mL of a VersaLyse solution and stained with antibodies. A de tailed description of the sorting and gating procedure is available in Ref. [2]. After sorting, the suspension was centrifuged for 10 min at 2,000 g.

**RNA separation and next-generation sequencing**

ExtractRNA reagent (900 µL) was added to the cells frozen in dry ice, and RNA isolation was performed according to the protocol. The RNA concentration was measured using the QuantiFluor® RNA System (Promega, USA). The quality of leukocyte RNA was determined on a TapeStation instrument (Agilent, USA). Only samples with an RNA Integrity Number (RIN) > 7 were used in the study. Small RNA li braries were constructed using the MGIEasy Small RNA Library Prep Kit V2.0 (BGI-940-000196-00). The prepared libraries were purified by electro phoresis in 6% polyacrylamide gel (PAGE) by cut ting a 100–130 bp band corresponding to a transcript of 17–47 nt in length. Sequencing was performed on a DNBSEQ-G400 instrument (BGI) using the DNBSEQ-G400RS High-throughput Sequencing Set (Small RNA FCL SE50) (BGI-1000016998).

**Bioinformatics and statistics**

A file 700–1,200 MB in size (11–20 million reads per f ile, with a Q30 score of 93–97) was obtained from each sample. Adapter trimming, miRNA annotation, and the search for novel miRNAs were performed using the Mirmaster 2.0 web service [11]. Annotation of known miRNAs was performed using MirBase [12] version 22.1. Raw data are presented in the Supplementary Materials. Nomenclature of the new miRNAs was performed using the built-in algorithm of the Mirmaster 2.0 web service. Graphs were plot ted in RStudio version 2023.09.1 + 494.pro2. The reli ability of the differences in the levels of both known and newly predicted miRNAs was analyzed using the Wilcoxon–Mann–Whitney test. The miRNA profiles were analyzed using the miRNA Enrichment Analysis and Annotation Tool (miEAA 2.1) [13]. To subject our data to miEAA, we used the Fold change-p-value ra tio with the following formula: –log10 (p) · sign(log2 (fold change)). Signaling pathways were annotated using miRTarBase (Release 9.0 beta) [14]. Only signaling pathways and targets with a Q-value < 0.05 were se lected for analysis. Prediction of novel miRNAs tar gets was executed using the miRDB service [15].

## RESULTS

**Fig. 1:**
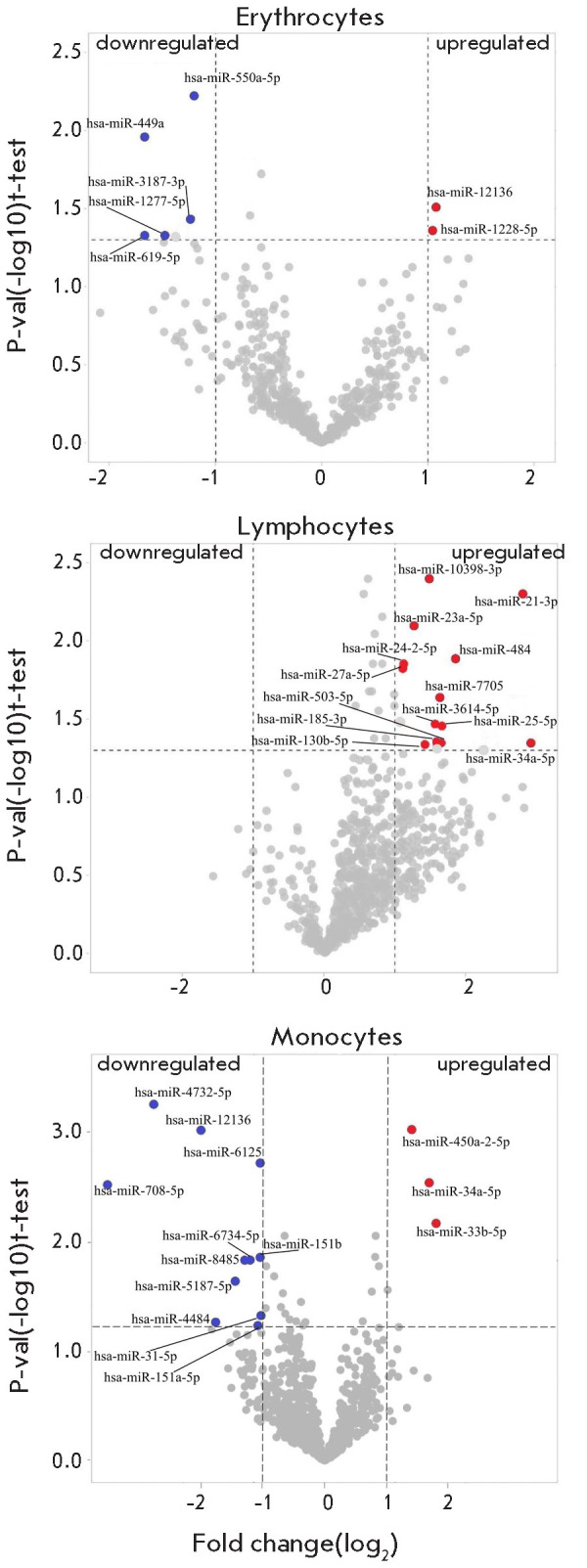
Differential expression of miRNAs in erythrocytes, lymphocytes, and monocytes. Lists of miRNAs with signifi cantly altered normalized expression levels compiled from the obtained data. These lists are presented in Table 2

We performed a NGS analysis for each sorted blood cell type separately: erythrocytes, lymphocytes, and monocytes. Analysis of the differential miRNA ex pression in erythrocytes revealed two upregulated and five downregulated miRNAs (Fig. 1, Table 2). All the statistically significant 13 miRNAs in lymphocytes appeared as upregulated (Fig. 1, Table 2). Monocytes had a more diverse pattern in terms of upregulation and downregulation: three upregulated and 11 down regulated (Fig. 1, Table 2).

**Table 2:** Differential expression of miRNAs in erythrocytes, lymphocytes, and monocytes during severe COVID-19

miRNA	Fold change	P-value	Expression
Erythrocytes			
hsa-miR-550a-5p	2.25	0.016	downregulated
hsa-miR-12136	2.60	0.016	upregulated
hsa-miR-449a	3.18	0.02	downregulated
hsa-miR-619-5p	3.18	0.021	downregulated
hsa-miR-1277-5p	2.36	0.032	downregulated
hsa-miR-1228-5p	2.11	0.032	upregulated
hsa-miR-3187-3p	2.30	0.036	downregulated
Lymphocytes			
hsa-miR-21-3p	6.99	0.029	upregulated
hsa-miR-23a-5p	3.62	0.029	upregulated
hsa-miR-27a-5p	2.18	0.029	upregulated
hsa-miR-24-2-5p	2.16	0.029	upregulated
hsa-miR-25-5p	3.11	0.029	upregulated
hsa-miR-484	3.17	0.029	upregulated
hsa-miR-34a-5p	7.58	0.029	upregulated
hsa-miR-185-3p	2.69	0.029	upregulated
hsa-miR-503-5p	2.72	0.029	upregulated
hsa-miR-3614-5p	3.08	0.029	upregulated
hsa-miR-130b-5p	2.47	0.029	upregulated
hsa-miR-10398-3p	2.80	0.029	upregulated
hsa-miR-7705	2.42	0.029	upregulated
Monocytes			
hsa-miR-4732-5p	6.89	0.008	downregulated
hsa-miR-12136	3.98	0.008	downregulated
hsa-miR-450a-2-3p	2.67	0.008	upregulated
hsa-miR-708-5p	11.62	0.008	downregulated
hsa-miR-34a-5p	3.21	0.008	upregulated
hsa-miR-6734-5p	2.34	0.008	downregulated
hsa-miR-8485	2.43	0.008	downregulated
hsa-miR-6125	2.07	0.012	downregulated
hsa-miR-33b-5p	3.52	0.016	upregulated
hsa-miR-151b	2.07	0.016	downregulated
hsa-miR-4484	3.42	0.032	downregulated
hsa-miR-31-5p	2.12	0.032	downregulated
hsa-miR-151a-5p	2.01	0.032	downregulated
hsa-miR-5187-5p	2.39	0.036	downregulated

**Evaluation of the signaling pathways altered by miRNAs in erythrocytes, lymphocytes, and monocytes**

**Fig. 2:**
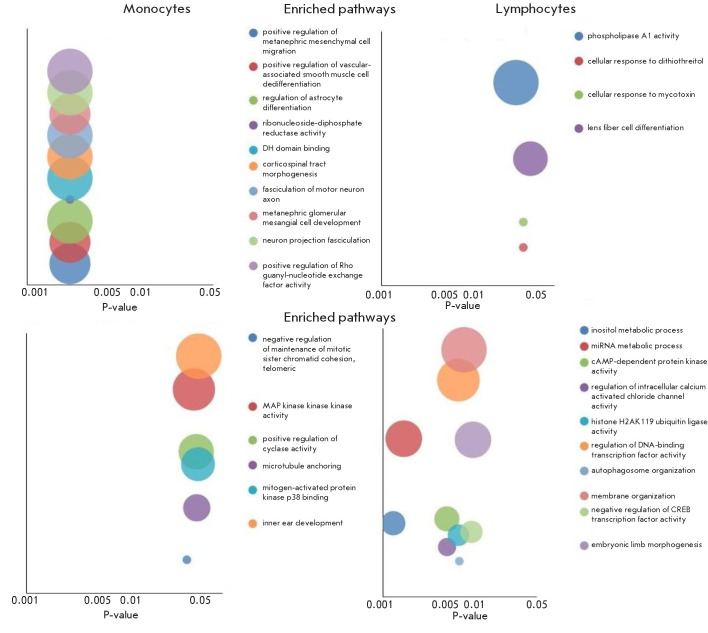
Evaluation of the major signaling pathways significantly (Q-value < 0.05) altered by miRNAs in monocytes and lymphocytes

To investigate the potential of changes (statistical en richment or depletion) in signaling pathways dur ing severe COVID-19, we performed a miRNA en richment analysis using the miEAA and miRTarBase (Gene Ontology) tools. The pathways with the small est Q-values are shown in Fig. 2. The complete list of pathways (Q-value < 0.05) is provided in the Supplementary Materials. These algorithms did not identify any signaling pathways whose profile had been significantly altered in erythrocytes during COVID-19. However, microRNAs were found to in f luence various signaling pathways in monocytes and lymphocytes.

**Identification of novel miRNAs with altered expression during severe COVID-19**

**Fig. 3:**
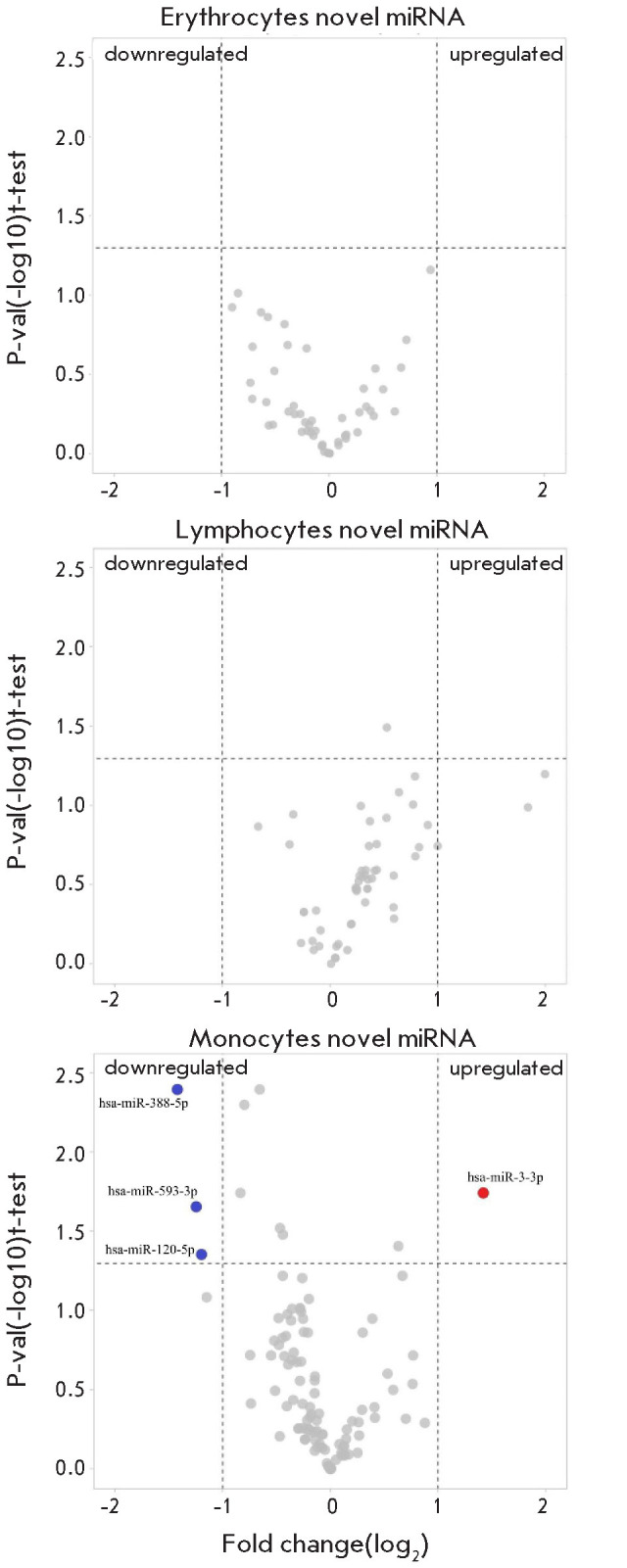
Predicted novel miRNA differential expression in erythrocytes, lymphocytes, and monocytes

The evaluation of novel miRNAs in the three cell pop ulations studied revealed that only four such miRNAs were present in monocytes. No significant changes in such miRNAs were detected in other cell types (Fig. 3). Table 3 summarizes the sequences of these microRNAs and their precursors.

**Table 3:** Predicted four novel miRNAs that alter their expression in monocytes during severe COVID-19

miRNA	Fold change	P-value	Expression	miRNA, nucleotidesequence	Pre-miRNA, nucleotide sequence
hsa-miR-3-3p	2.67	0.032	upregulated	GGGUGCGGGCCGGCGGGGUCCU	GACCUCGCCGUCCCGCCCGCCGCCUUCUGCGUCGCGGGUGCGGGCCGGCGGGGUCCU
hsa-miR-120-5p	2.29	0.01	downregulated	UGGGGGAGGAGGAAGAGGAGA	UGGGGGAGGAGGAAGAGGAGAUGGGGAGGCAGGUGAGCCUGACCAAGCAGCCUGCUCCCUUUCUCCCUCCCCUUCCCCCUC
hsa-miR-593-3p	2.37	0.032	downregulated	UUUGGGGAUUCUAAGAGGAAG	AACUCUUAGAAUCCCCAAAGCAUUCUGUGAAGUGGUUUGGGGAUUCUAAGAGGAAG
hsa-miR-388-5p	2.67	0.008	downregulated	GUCCCAGCAACUCAGGAGGCUAAGG	GUCCCAGCAACUCAGGAGGCUAAGGUGGGAGGAUCACUUGAGCCCAGGAGUUCUGGGCUG

In order to assess how these miRNAs may af fect signaling in cells, we attempted to locate pos sible target genes using the miRDB service [15]. The most confident targets (score > 95) are pre sented in Table 4. A complete list of possible tar gets (score > 50) is provided in the Supplementary Materials. No high-confidence targets (score > 95) were identified for hsa-miR-3-3p.

**Table 4:** The potentially important target genes of the predicted miRNAs

Predicted miRNA	Target gene, conditional prediction confidence, a.u.	Target gene, symbol	Target gene-encoded protein
hsa-miR-120-5p	100	MECP2	methyl-CpG binding protein 2
	100	PPP1R9B	Protein phosphatase 1 regulatory subunit 9B
	99	SLC6A17	Solute carrier family 6 member 17
	99	WIZ	WIZ zinc finger
	98	NFIX	Nuclear factor I X
	98	CASTOR2	Cytosolic arginine sensor for mTORC1 subunit 2
	98	NR1D1	Nuclear receptor subfamily 1 group D member 1
	97	NLGN2	Neuroligin 2
	97	ELK1	ELK1, ETS transcription factor
	97	SHISAL1	Shisa like 1
	96	CAMK1D	Calcium/calmodulin dependent protein kinase ID
	96	ACTB	Actin beta
	96	HEYL	Hes related family bHLH transcription factor with YRPW motif-like
	96	SRF	Serum response factor
	96	EPB41L1	Erythrocyte membrane protein band 4.1 like 1
hsa-miR-593-3p	98	CEP135	Centrosomal protein 135
	98	ELK1	ELK1, ETS transcription factor
	98	ZNF629	Zinc finger protein 629
	97	SUSD2	Sushi domain containing 2
	97	ARGFX	Arginine-fifty homeobox
	97	IGF2	Insulin-like growth factor 2
	97	ZDHHC8	Zinc finger DHHC-type containing 8
	97	BCAM	Basal cell adhesion molecule (Lutheran blood group)
	96	GDF11	Growth differentiation factor 11
	96	TAB3	TGF-beta activated kinase 1 (MAP3K7) binding protein 3
	96	PKNOX2	PBX/knotted 1 homeobox 2
	96	ZBTB39	Zinc finger and BTB domain containing 39
hsa-miR-388-5p	99	STK4	Serine/threonine kinase 4
	98	FOXK1	Forkhead box K1
	98	PCBP1	poly(rC) binding protein 1
	97	BSDC1	BSD domain containing 1
	97	LRTOMT	Leucine rich transmembrane and O-methyltransferase domain containing
	96	KIAA0930	KIAA0930
	96	TUB	Tubby bipartite transcription factor
	96	NRP1	Neuropilin 1
	96	CRP	C-reactive protein

## DISCUSSION

A few important remarks are in order prior to dis cussing the result’s validity. Most of the changes in the expression profiles are most likely unrelated to the effects of hypoxia, due to the fact that the O_2_ saturation was above 93% in all the patients with se vere COVID-19, which is not significant enough to have any effect on blood cells. These changes are not associated with serious comorbidities in COVID-19 patients, since no such patients were added to the sample. Furthermore, follow-up sequencing was performed to assess the validity of the changes in miRNA concentrations. As a result, expression levels of less than ten reads were shown to be poorly re producible by such sequencing. Hence, we considered significant changes in levels only for those miRNAs where at least one group (control or COVID-19) had median levels of a particular miRNA above ten reads. All the miRNAs reported in this study as having sig nificant differences met our stated criterion.

The mechanism of miRNA profile changes in erythrocytes is not fully understood. Mature eryth rocytes lack nuclei [16] and are therefore unable to synthesize pre-miRNA. Therefore, expression reg ulation should be carried out at the stage of either pre-miRNA or miRNA excision. The expression pro file can also be regulated at the stage of an immature erythrocyte possessing a nucleus. However, the latter mechanism is unlikely, since the COVID-19-induced cytokine storm develops rapidly, before any substan tial renewal of peripheral blood erythrocytes. Given the fact that the average lifetime of an erythrocyte is 120 days [17], during severe COVID-19 it is likely that their lifespan will be shortened by rapid hemolysis and erythrocyte renewal. It is still not exactly clear how quickly these cells are renewed during a SARS CoV-2 infection. There are few examples of condi tions under which the miRNA profile in erythrocytes is altered, something that is typically due to chronic diseases or other long-term adverse influences on the organism. It has been demonstrated that the miRNA expression pattern changes during sickle cell anemia [7], paroxysmal nocturnal hemoglobinuria [8], as well as Parkinson’s disease [9]. Additionally, people living in high-altitude mountain regions also exhibit chang es in miRNA expression [18]. miRNAs contained in erythrocytes cannot influence the synthetic ability of these cells, since erythrocytes lack ribosomes and, therefore, lack translation and miRNA-mediated si lencing. However, it has been shown that erythrocytes can release vesicles that circulate in the blood. The contents of these vesicles can enter another cell, and miRNA will change the gene expression in that cell.

When observing the significant hits in erythrocyte miRNAs, we found only two miRNAs to be upreg ulated: miR-1228-5p and hsa-miR-12136. Both have been identified as differentially expressed miRNAs in various studies related to COVID-19. In particu lar, miR-1228-5p was found among 246 differential ly expressed miRNAs in plasma exosomes from pa tients, an indication of its potential involvement in the disease process and response to the infection [19]. hsa-miR-12136 was among the top ten differentially expressed and upregulated miRNAs in patients with COVID-19. Additionally, a ROC analysis demonstrat ed that the levels of hsa-miR-12136, along with other miRNAs, can help differentiate hospitalized COVID-19 patients from healthy uninfected controls with high efficiency [20]. This fact aligns with our observations of hsa-miR-12136 demonstrating the second-highest upregulated fold change state among seven other dif ferentially expressed miRNAs. On the other hand, the downregulated hsa-miR-449a demonstrated signifi cant differential expression and statistical significance. hsa-miR-449a has been identified as a tumor suppres sor in various cancers, including neuroblastoma and endometrial cancer [21]. It is known to inhibit cancer cell proliferation by inducing cell differentiation and causing cell cycle arrest. For instance, in neuroblastoma, hsa-miR-449a overexpression leads to the dif ferentiation of cancer cells and downregulation of key cell cycle regulators such as CDK6 and LEF1 [22].

In lymphocytes, we observed the upregulation of several miRNAs. One of these was hsa-miR-21-3p. It has previously been shown that six miRNAs, includ ing miR-21-3p, can directly bind to the RNA of all human coronavirus genomes, including SARS-CoV-2, and regulate viral gene expression. Among these, miR-21-3p exhibited the highest binding affinity to the human coronavirus genome [23]. Our data align with those of previously published research indicating that hsa-miR-21-3p is significantly upregulated during the SARS-CoV-2 infection. This upregulation is asso ciated with a delayed immune response, which may foster viral survival and replication. Specifically, hsa miR-21-3p has been shown to interact with the viral polyprotein 1a mRNA, a conserved feature across hu man coronaviruses [24, 25]. Another significantly up regulated miRNA was hsa-miR-9-5p, which has been identified as a miRNA that can target the 3’-untrans lated region (3’UTR) of the ACE2 gene. The ACE2 protein (angiotensin-converting enzyme 2) is essential for SARS-CoV-2 entry into host cells. By targeting ACE2, hsa-miR-9-5p may potentially influence suscep tibility to the infection and the severity of COVID-19 symptoms [26]. Furthermore, Haldar et al. reported hsa-miR-23a-5p to be associated with the host pro tein genes involved in the SARS-CoV-2 infection; SERPING1 in particular [27]. The SERPING1 gene encodes the C1 inhibitor (C1-INH) protein, a crucial member of the serpin superfamily of serine prote ase inhibitors [28]. C1-INH helps control inflammation by inhibiting plasma kallikrein and factor XIIa, both of which are involved in the production of bradyki nin, a peptide that increases blood vessel permeability and promotes inflammatory responses [29]. By bind ing to these proteins, C1-INH prevents excessive bra dykinin production, thereby regulating fluid transport into tissues during inflammatory responses [30]. The bradykinin activation theory is one of the most cred ible hypotheses seeking to explain the cardiovascular complications that occur during severe COVID-19 [31]. Our data on the involvement of hsa-miR-23a-5p in the pathogenesis of severe COVID-19 agree with the aforementioned studies [27].

Among the three upregulated miRNAs, hsa-miR-33b-5p is worthy of note. miR-33b is known to influence various cellular functions that are re lated to the immune response and inflammation. It is involved in the regulation of pro-inflammatory cy tokine production and macrophage polarization. For example, miR-33b was shown to modulate expres sion of the genes involved in inflammatory pathways, thereby affecting monocyte survival and functioning during inflammatory responses [32, 33]. Furthermore, another microRNA identified in our study of mono cytes, hsa-miR-151a-5p, was shown to bind directly to SARS-CoV-2 RNA transcripts [26], specifically target ing the spike protein gene [34]. hsa-miR-151a-5p was implicated in the modulation of the inflammatory re sponse during the SARS-CoV-2 infection [35]. As stat ed by various researchers, the typical dysregulation of the immune response observed during COVID-19 tends to be associated with changes in the expres sion levels of several miRNAs, hsa-miR-151a-5p being among them [35, 36].

In addition to studying the differential expres sion of miRNA in erythrocytes, lymphocytes, and monocytes, we subjected our sets of miRNA to a miRNA Enrichment Analysis (miEAA), which al lowed us to analyze the various pathways in which the miRNAs are involved (Fig. 2). The miEAA oper ates in GeneOntology (GO) terms [37]. Erythrocytes demonstrated no statistically significantly enriched or depleted pathways. This result derives from the fact that a mature erythrocyte has neither a strong signal transduction system nor serious transcriptional activ ity. A 2020 study of miRNA pathways showed that miR-4732-3p targets components of the TGF-β sig naling pathway (SMAD2 and SMAD4) [38], which are involved in erythropoiesis and promote cell prolifera tion during erythroid differentiation. A 2024 study of hematopoiesis regulation demonstrated how miR-7145 enhances erythropoiesis, while inhibiting myeloid pro genitor cell differentiation through the JAK1/STAT3 signaling pathway [39]. Its expression correlates with that of GATA1, a key transcription factor in erythro cyte development. Nevertheless, the aforementioned miRNAs are involved in the erythrocyte progenitor stages, which allows us to suggest that the discov ery of these miRNAs in mature erythrocytes is more likely to be evidence of processes occurring during erythropoiesis. Hence, the observed absence of signal ing pathways in mature erythrocytes can be regarded as confirmation of low or absent synthetic activity. Regarding statistically significantly enriched pathways in monocytes, one of the most credible pathways ap peared to be the positive regulation of metanephric mesenchymal cell migration. Certain miRNAs have been identified as positive regulators of cell migration. For example, miR-200 family members are known to influence epithelial-to-mesenchymal transition (EMT), a process relevant to mesenchymal cell migration [40]. Given that severe COVID-19 leads to immune hy peractivation and immunological dysfunction [41], it is reasonable to posit that the observed enrichment in this pathway is a consequence of these process es. Positive regulation of vascular-associated smooth muscle cell (VSMC) differentiation is important for vascular development and remodeling. The miRNAs involved in VSMC are miR-143/145. This miRNA clus ter is crucial for VSMC differentiation. It promotes the expression of contractile proteins, while inhibiting pathways that lead to dedifferentiation and prolifera tion. The loss of miR-143/145 leads to impaired VSMC differentiation and contributes to vascular pathologies. Additionally, these miRNAs are positively regulated by the serum response factor (SRF) and myocardin, which are critical in promoting VSMC differentiation by suppressing the factors that inhibit the process [42]. Regulation of astrocyte differentiation (another pathway that we have identified) is important for the development of the central nervous system (CNS). Key regulatory factors include RNF20 [43] – an E3 ubiquitin ligase; TAZ (WW domain-containing tran scription regulator 1) and YAP (Yes-associated pro tein) – transcriptional co-activators involved in the Hippo signaling pathway [44]; and the transcription factor PITX1 [45], which controls astrocyte differen tiation by regulating SOX9 expression. The involve ment of miRNAs in this process had not previously been demonstrated. Recent studies have also high lighted the regulatory role of miRNAs in modulating ribonucleoside-diphosphate reductase (RNR) activ ity, particularly through the regulation of its RRM2 subunit. hsa-miR-125b-5p and hsa-miR-30a-5p have been shown to negatively correlate with the RRM2 expression in various cancer types. Their regulation suggests that these miRNAs may play a role in the maintenance of appropriate dNTP levels by modulat ing RNR activity [46]. DH domain binding emerged as another enriched pathway. MicroRNAs interact with RNA-binding proteins (RBPs) that contain specific do mains, such as DH. By modulating miRNA levels and activity, RBPs can influence target gene expression, impacting cellular functions such as proliferation, dif ferentiation, and response to stress. The movement of miRNAs into extracellular vesicles is mediated by specific RBPs, and, consequently, this process plays an important role in intercellular signaling and tis sue interactions [47]. Corticospinal tract (CST) mor phogenesis is another process shown to be regulated by miRNAs. miRNAs often work in conjunction with RNA-binding proteins, which facilitate their loading into the RNA-induced silencing complex (RISC). The miR-34/449 family has been shown to fine-tune the expression of the genes critical for spinal interneuron development. Studies involving mutant mice lacking miR-34/449 revealed a notable disruption in the ge netic profiles of spinal cord neurons, indicating that these miRNAs are essential for the proper circuit for mation necessary for motor control [48]. Furthermore, miRNAs are key regulatory molecules in motor neu ron axon fasciculation [49]. In motor neurons, miRNAs are involved in several developmental processes. For example, modifying the miR-9 expression has been shown to affect motor neuron subtype specification and spinal cord development. Additionally, miR-17-3p regulates the stability of Olig2 transcription factor mRNA, which is critical for spinal motor neuron dif ferentiation [50]. miRNAs also play crucial roles in the early stages of kidney development, particularly in the differentiation of nephron progenitor cells, which give rise to glomerular structures. Specific miRNAs, such as members of the miR-30 family, have been shown to target key transcription factors like Lhx1, which is vital for nephrogenesis [51, 52]. Furthermore, neuron fasciculation is significantly influenced by miRNAs. Several specific miRNAs have been impli cated in the fasciculation process. In particular, miR-8 has been shown to regulate the expression of the cell adhesion molecules critical for synapse formation and may also play a role in axon guidance during synap togenesis in Drosophila [53].

The positive regulation of RhoGEFs by miRNAs is particularly relevant in cancer biology. Aberrant expression of specific miRNAs can lead to enhanced RhoGEF activity, promoting cancer cell migration and invasion. This has been observed in various cancer types where dysregulated miRNA profiles correlate with increased metastatic potential due to modified Rho GTPase signaling pathways [54].

The inner ear development pathway is one of the most prominent signaling pathways in monocytes. The miR-183/96/182 cluster is one of the groups of miRNAs whose functions and expression are best-studied in inner ear development. Studies us ing animal models have shown that knockout of these miRNAs causes severe damage to hair cell develop ment and hearing loss [55, 56]. Additionally, several miRNAs that regulate the MAPK signaling pathway were identified in monocytes. In particular, miR-203 regulates BCR-ABL levels and inhibits cell prolifera tion in chronic myeloid leukemia (CML) by silenc ing the mRNA of MAPK pathway components [57]. It is also known that miR-155 is involved in the reg ulation of the SOS and KRAS proteins, influencing MAPK/ERK pathway activity. Furthermore, miR-19a regulates RAF1 and other components of the MAPK cascade, affecting the overall signaling dynam ics of this pathway. Meanwhile, miR-128 affects c Met/PI3K/AKT signaling, which is linked to MAPK pathways, particularly in lung cancer [58].

It is known that some miRNAs can directly target mRNAs encoding cyclases, leading to their degrada tion or translational repression. For instance, miR-282 has been identified as a regulator of adenylate cy clase in the nervous system, suggesting modulation of pathways associated with cAMP signals important for neuronal function [59].

The miRNA sets in lymphocytes demonstrated less involvement in GO terms pathways; however, they seem to be more specific. Among the enriched GO terms, Phospholipase A1 (PLA1) is an enzyme that hydrolyzes phospholipids, playing a crucial role in lipid metabolism and cell signaling within lympho cytes. The regulation of the PLA1 activity is linked to the function of some miRNAs. For instance, the miR-17~92 cluster regulates B-cell survival by tar geting the mRNAs of pro-apoptotic factors such as BIM [60]. miRNAs are crucial for the maturation of T and B cells, ensuring that autoreactive cells are eliminated during development to prevent autoim munity [60]. During T cell activation, specific miRNAs such as miR-155 are upregulated, enhancing effector functions like cytokine production. Conversely, oth er miRNAs can suppress activation to maintain ho meostasis [61, 62]. These miRNAs are likely to serve as mediators in the immunological misfiring that oc curs during the cytokine storm induced by severe COVID-19 [41]. Cellular response to dithiothreitol (DTT) is another signaling cascade associated with the miRNAs identified in our study. It has previous ly been shown that miR-101 regulates SEL1L ex pression, which is involved in endoplasmic reticulum (ER) stress response. Under conditions of ER stress, for example induced by DTT, regulation of SEL1L by miR-101 may influence neuronal cell death path ways [63, 64].

Possible target genes controlled by the newly pre dicted miRNAs in monocytes are also of peculiar in terest. Thus, hsa-miR-388-5p presumably controls the synthesis of C-reactive protein, one of the pro teins of acute inflammation (Table 4). This protein is synthesized by liver cells. However, it is possible that monocytes secrete this miRNA in extracellular ves icles with the purpose of signaling to the liver cells. In the investigated system, the hsa-miR-388-5p level in monocytes during severe COVID-19 is decreased compared to that in healthy donors. This may be an additional factor increasing the CRP blood level in patients with severe COVID-19.

Summarizing our work, we would like to emphasize that its main result is that the spectrum of miRNAs being altered in monocytes, erythrocytes, and lym phocytes during severe COVID-19 has been identified. This can be used to search for potential predictors of severe COVID-19. Additionally, our work would help elucidate the changes in the molecular mechanisms in blood cells not only during COVID-19-induced cy tokine storm, but also during other viral infections.

## References

[1] Artamonov AA., Kondratov KA., Bystritsky EA. (2024). Changes in the Repertoire of tRNA-Derived Fragments in Different Blood Cell Populations.. Life (Basel)..

[2] Kondratov KA., Artamonov AA., Nikitin YV. (2024). Revealing differential expression patterns of piRNA in FACS blood cells of SARS-CoV-2 infected patients.. BMC Med Genomics..

[3] Baulina NM., Kulakova OG., Favorova OO. (2016). MicroRNAs: The Role in Autoimmune Inflammation.. Acta Naturae..

[4] Iqbal N., Kumar P. (2022). Integrated COVID-19 Predictor: Differential expression analysis to reveal potential biomarkers and prediction of coronavirus using RNA-Seq profile data.. Comput Biol Med..

[5] Aznaourova M., Schmerer N., Janga H. (2022). Single-cell RNA sequencing uncovers the nuclear decoy lincRNA PIRAT as a regulator of systemic monocyte immunity during COVID-19.. Proc Natl Acad Sci U S A..

[6] Chattopadhyay P., Mehta P., Soni J., Tardalkar K., Joshi M., Pandey R. (2024). Cell-specific housekeeping role of lncRNAs in COVID-19-infected and recovered patients.. NAR Genom Bioinform..

[7] Chen SY., Wang Y., Telen MJ., Chi JT. (2008). The genomic analysis of erythrocyte microRNA expression in sickle cell diseases.. PLoS One..

[8] Chi JT., Sangokoya C., De Castro CM. (2007). MicroRNA Expression in Red Blood Cells from Patients with PNH.. Blood..

[9] Shaheen N., Shaheen A., Osama M., Nashwan AJ., Bharmauria V., Flouty O. (2024). MicroRNAs regulation in Parkinson’s disease, and their potential role as diagnostic and therapeutic targets.. NPJ Parkinsons Dis..

[10] Wu Y., Leyk S., Torabi H. (2023). Plasmodium falciparum infection reshapes the human microRNA profiles of red blood cells and their extracellular vesicles.. iScience..

[11] Fehlmann T., Kern F., Laham O. (2021). miRMaster 2.0: multi-species non-coding RNA sequencing analyses at scale.. Nucleic Acids Res..

[12] Kozomara A., Birgaoanu M., Griffiths-Jones S. (2019). miRBase: from microRNA sequences to function.. Nucleic Acids Res..

[13] Backes C., Khaleeq QT., Meese E., Keller A. (2016). miEAA: microRNA enrichment analysis and annotation.. Nucleic Acids Res..

[14] Huang HY., Lin YC., Cui S. (2022). miRTarBase update 2022: an informative resource for experimentally validated miRNA-target interactions.. Nucleic Acids Res..

[15] Chen Y., Wang X. (2020). miRDB: an online database for prediction of functional microRNA targets.. Nucleic Acids Res..

[16] Zhang ZW., Cheng J., Xu F. (2011). Red blood cell extrudes nucleus and mitochondria against oxidative stress.. IUBMB Life..

[17] Thiagarajan P., Parker CJ., Prchal JT. (2021). How Do Red Blood Cells Die?. Front Physiol..

[18] Sun L., Fan F., Li R. (2018). Different Erythrocyte MicroRNA Profiles in Low- and High-Altitude Individuals.. Front Physiol..

[19] Chen XM., Yao DN., Wang MJ. (2022). Deep Sequencing of Plasma Exosomal microRNA Level in Psoriasis Vulgaris Patients.. Front Med (Lausanne)..

[20] Franco S., Mateu L., Pluvinet R. (2024). Altered Plasma microRNA Signature in Hospitalized COVID-19 Patients Requiring Oxygen Support.. Microorganisms..

[21] Zhao Z., Ma X., Sung D. (2015). microRNA-449a functions as a tumor suppressor in neuroblastoma through inducing cell differentiation and cell cycle arrest.. RNA Biol..

[22] Ye W., Xue J., Zhang Q. (2014). MiR-449a functions as a tumor suppressor in endometrial cancer by targeting CDC25A.. Oncol Rep..

[23] Nersisyan S., Engibaryan N., Gorbonos A., Kirdey K., Makhonin A., Tonevitsky A. (2020). Potential role of cellular miRNAs in coronavirus-host interplay.. PeerJ..

[24] Arghiani N., Nissan T., Matin MM. (2021). Role of microRNAs in COVID-19 with implications for therapeutics.. Biomed Pharmacother..

[25] Zhang WC., Skiados N., Aftab F. (2022). MicroRNA-21 guide and passenger strand regulation of adenylosuccinate lyase-mediated purine metabolism promotes transition to an EGFR-TKI-tolerant persister state.. Cancer Gene Ther..

[26] Yang CY., Chen YH., Liu PJ., Hu WC., Lu KC., Tsai KW. (2022). The emerging role of miRNAs in the pathogenesis of COVID-19: Protective effects of nutraceutical polyphenolic compounds against SARS-CoV-2 infection.. Int J Med Sci..

[27] Haldar A., Yadav KK., Singh S., Yadav PK., Singh AK. (2022). In silico analysis highlighting the prevalence of BCL2L1 gene and its correlation to miRNA in human coronavirus (HCoV) genetic makeup.. Infect Genet Evol..

[28] Gösswein T., Kocot A., Emmert G. (2008). Mutational spectrum of the C1INH (SERPING1) gene in patients with hereditary angioedema.. Cytogenet Genome Res..

[29] Wouters D., Wagenaar-Bos I., van Ham M., Zeerleder S. (2008). C1 inhibitor: just a serine protease inhibitor? New and old considerations on therapeutic applications of C1 inhibitor.. Expert Opin Biol Ther..

[30] Drouet C., López-Lera A., Ghannam A. (2022). SERPING1 Variants and C1-INH Biological Function: A Close Relationship With C1-INH-HAE.. Front Allergy..

[31] Wilczynski SA., Wenceslau CF., McCarthy CG., Webb RC. (2021). A Cytokine/Bradykinin Storm Comparison: What Is the Relationship Between Hypertension and COVID-19?. Am J Hypertens..

[32] Sampath P., Periyasamy KM., Ranganathan UD., Bethunaickan R. (2021). Monocyte and Macrophage miRNA: Potent Biomarker and Target for Host-Directed Therapy for Tuberculosis.. Front Immunol..

[33] Ubilla CG., Prado Y., Angulo J. (2021). MicroRNA-33b is a Potential Non-Invasive Biomarker for Response to Atorvastatin Treatment in Chilean Subjects With Hypercholesterolemia: A Pilot Study.. Front Pharmacol..

[34] Sardar R., Satish D., Birla S., Gupta D. (2020). Integrative analyses of SARS-CoV-2 genomes from different geographical locations reveal unique features potentially consequential to host-virus interaction, pathogenesis and clues for novel therapies.. Heliyon..

[35] Liang Y., Fang D., Gao X. (2023). Circulating microRNAs as emerging regulators of COVID-19.. Theranostics..

[36] Garnier N., Pollet K., Fourcot M. (2022). Altered microRNA expression in severe COVID-19: Potential prognostic and pathophysiological role.. Clin Transl Med..

[37] Ashburner M., Ball CA., Blake JA. (2000). Gene ontology: tool for the unification of biology. The Gene Ontology Consortium.. Nat Genet..

[38] Sun L., Yu Y., Niu B., Wang D. (2020). Red Blood Cells as Potential Repositories of MicroRNAs in the Circulatory System.. Front Genet..

[39] Xu Y., Guo R., Huang T., Guo C. (2024). miRNA-7145-cuedc2 axis controls hematopoiesis through JAK1/STAT3 signaling pathway.. Cell Death Discov..

[40] Nalbant E., Akkaya-Ulum YZ. (2024). Exploring regulatory mechanisms on miRNAs and their implications in inflammation-related diseases.. Clin Exp Med..

[41] Lucas C., Wong P., Klein J. (2020). Longitudinal analyses reveal immunological misfiring in severe COVID-19.. Nature.

[42] Wang G., Jacquet L., Karamariti E., Xu Q. (2015). Origin and differentiation of vascular smooth muscle cells.. J Physiol..

[43] Liang Q., Xia W., Li W., Jiao J. (2018). RNF20 controls astrocytic differentiation through epigenetic regulation of STAT3 in the developing brain.. Cell Death Differ..

[44] Chen J., Tsai YH., Linden AK., Kessler JA., Peng CY. (2024). YAP and TAZ differentially regulate postnatal cortical progenitor proliferation and astrocyte differentiation.. J Cell Sci..

[45] Byun JS., Oh M., Lee S. (2020). The transcription factor PITX1 drives astrocyte differentiation by regulating the SOX9 gene.. J Biol Chem..

[46] Wu L., Yin L., Ma L. (2022). Comprehensive bioinformatics analysis of ribonucleoside diphosphate reductase subunit M2(RRM2) gene correlates with prognosis and tumor immunotherapy in pan-cancer.. Aging (Albany NY)..

[47] Hobor F., Dallmann A., Ball NJ. (2018). A cryptic RNA-binding domain mediates Syncrip recognition and exosomal partitioning of miRNA targets.. Nat Commun..

[48] Chang SH., Su YC., Chang M., Chen JA. (2021). MicroRNAs mediate precise control of spinal interneuron populations to exert delicate sensory-to-motor outputs.. Elife..

[49] Iyer AN., Bellon A., Baudet ML. (2014). microRNAs in axon guidance.. Front Cell Neurosci..

[50] Kye MJ., Gonçalves Ido C. (2014). The role of miRNA in motor neuron disease.. Front Cell Neurosci..

[51] Trionfini P., Benigni A. (2017). MicroRNAs as Master Regulators of Glomerular Function in Health and Disease.. J Am Soc Nephrol..

[52] Marciano DK. (2019). Mesangial Cells: The Tuft Guys of Glomerular Development.. J Am Soc Nephrol..

[53] Lu CS., Zhai B., Mauss A., Landgraf M., Gygi S., Van Vactor D. (2014). MicroRNA-8 promotes robust motor axon targeting by coordinate regulation of cell adhesion molecules during synapse development.. Philos Trans R Soc Lond B Biol Sci..

[54] Humphries BA., Wang Z., Yang C. (2020). MicroRNA Regulation of the Small Rho GTPase Regulators-Complexities and Opportunities in Targeting Cancer Metastasis.. Cancers (Basel)..

[55] Mittal R., Liu G., Polineni SP., Bencie N., Yan D., Liu XZ. (2019). Role of microRNAs in inner ear development and hearing loss.. Gene..

[56] Geng R., Furness DN., Muraleedharan CK. (2018). The microRNA-183/96/182 Cluster is Essential for Stereociliary Bundle Formation and Function of Cochlear Sensory Hair Cells.. Sci Rep..

[57] Chakraborty C., Sharma AR., Patra BC., Bhattacharya M., Sharma G., Lee SS. (2016). MicroRNAs mediated regulation of MAPK signaling pathways in chronic myeloid leukemia.. Oncotarget..

[58] Maharati A., Zanguei AS., Khalili-Tanha G., Moghbeli M. (2022). MicroRNAs as the critical regulators of tyrosine kinase inhibitors resistance in lung tumor cells.. Cell Commun Signal..

[59] Wu P., Jiang X., Guo X., Li L., Chen T. (2016). Genome-Wide Analysis of Differentially Expressed microRNA in Bombyx mori Infected with Nucleopolyhedrosis Virus.. PLoS One..

[60] Simpson LJ., Ansel KM. (2015). MicroRNA regulation of lymphocyte tolerance and autoimmunity.. J Clin Invest..

[61] Rodríguez-Galán A., Fernández-Messina L., Sánchez-Madrid F. (2018). Control of Immunoregulatory Molecules by miRNAs in T Cell Activation.. Front Immunol..

[62] Podshivalova K., Salomon DR. (2013). MicroRNA regulation of T-lymphocyte immunity: modulation of molecular networks responsible for T-cell activation, differentiation, and development.. Crit Rev Immunol..

[63] Omura T., Nomura L., Watanabe R. (2021). MicroRNA-101 Regulates 6-Hydroxydopamine-Induced Cell Death by Targeting Suppressor/Enhancer Lin-12-Like in SH-SY5Y Cells.. Front Mol Neurosci..

[64] Belkozhayev A., Niyazova R., Kamal MA., Ivashchenko A., Sharipov K., Wilson CM. (2024). Differential microRNA expression in the SH-SY5Y human cell model as potential biomarkers for Huntington’s disease.. Front Cell Neurosci..

